# The Efficacy of a Gambling Prevention Program Among High-School Students

**DOI:** 10.1007/s10899-019-09908-2

**Published:** 2019-11-11

**Authors:** Filipa Calado, Joana Alexandre, Liah Rosenfeld, Rafaela Pereira, Mark D. Griffiths

**Affiliations:** 1 Department of Psychology, Nottingham Trent University, 50 Shakespeare Street, Nottingham, NG1 4FQ UK; 2 Department of Psychology, ISCTE – CIS/IUL - Lisbon University Institute, Avenida das Forças Armadas, 1649-026 Lisbon, Portugal

**Keywords:** Youth gambling, Adolescent gambling, Gambling prevention, Problem gambling, Gambling intervention program

## Abstract

Youth problem gambling has become an emergent public health issue, and adolescents constitute a vulnerable age group for the development of gambling-related problems. Although there is research concerning the risk factors of youth problem gambling, rigorous evaluations of the effectiveness of preventive initiatives is still rare. The present study evaluated the efficacy of an integrative intervention to prevent youth problem gambling based on a multidimensional set of factors including gambling-related knowledge, misconceptions, attitudes, gambling frequency, amount of money spent, total hours spent gambling per week, and sensation seeking. A pre- and post-test design was performed with 111 Portuguese high-school students randomly assigned to two groups (experimental and control). The findings demonstrated that the intervention was effective in improving correct knowledge about gambling, reducing misconceptions and attitudes, and in decreasing the total hours spent gambling per week. The intervention was also effective in reducing the number of at-risk/problem gamblers during the study period. Furthermore, these findings were stable after a 6-week follow-up. Overall, the intervention program appeared to be effective in correcting some gambling-related behaviours, and provides suggestions for future interventions.

## Introduction

Although gambling is often conceptualized as an adult activity, research has consistently shown that problem gambling is an emergent public health issue among adolescents and emerging adults (Calado et al. 2017a), and is part of a broader constellation of other risk behaviours (antisocial, risk-taking, delinquent behaviours) at this developmental stage—particularly for males. Youth problem gambling can bring about severe negative consequences both in the short-term and in the long-term, such as mental health concerns (e.g., mood disorders, anxiety disorders), and behavioural problems (e.g., Kourgiantakis et al. 2016).

In addition, a growing body of research suggests that individuals with gambling-related problems start gambling in adolescent years (Carbonneau et al. 2015; Burge et al. 2006). Furthermore, due to limited developed cognitive ability, adolescents are more susceptible to gambling activities (Oh et al. 2017), which can lead to higher rates of problem gambling in comparison with adults. Therefore, the development of preventive initiatives that target this age group assumes particular importance. However, scientific knowledge of preventive initiatives and its empirical evaluation are still scarce, and in the areas of public health, prevention is recognised to be as crucial as treatment in reducing harm (Dickson-Gillespie et al. 2008).

In terms of prevention frameworks, the literature has identified three types of prevention initiatives for this particular problem: primary, secondary and tertiary (see Kourgiantakis et al. 2016). The former includes activities targeted to youth populations who not gamble to prevent gambling problems from arising in the first place, with the goal of increasing awareness of the risks involved and consequences associated with problem gambling behaviours. Some data suggests that 80% of youth are included in this group (Messerlian et al. 2005). Secondary prevention is designed to develop initiatives for children at-risk, in order to reduce the likelihood of developing severe problem gambling behaviours. Finally, tertiary prevention target adolescents that already show signs and symptoms of disordered gambling and mainly includes treatment programs (Kourgiantakis et al. 2016). A large body of evidence in the field of general youth prevention programs for social and behavioural problems have found that primary prevention efforts have the dual benefits of enhancing competencies as well as in reducing internalizing and externalizing problems (Durlak and Wells 1997; Greenberg et al. 2003). In addition, among preventive gambling initiatives, primary prevention programs provide a low-cost tool to target individuals who may not yet have engaged in the behaviour, but who are in an age group which is particularly susceptible for the development of problem gambling (Williams et al. 2010). In fact, the majority of prevention programs are classified in primary prevention programs, and most of them are considered to be within the category of school-based prevention programs (Huic et al. 2017). School-based programs are the most frequent prevention initiatives, and constitute a relevant part of an overall prevention strategy as they provide an intervention to an age group who is vulnerable and can prevent an escalation of problem behaviours into adulthood (Williams et al. 2010; Oh et al. 2017).

In order to achieve success utilizing preventive initiatives, risk and protective factors related to gambling are essential components in designing prevention programs. St-Pierre and Derevensky (2016) classified the school-based gambling-specific prevention programs into two broad categories: (1) psychoeducational prevention programs and (2) comprehensive psychoeducational and skills training prevention programs. Both types of preventive initiatives aim to increase the accurate knowledge of gambling odds, and to improve maladaptive gambling related cognitions and misconceptions. In fact, it has been found that lack of gambling knowledge can contribute to the development of problem gambling (Blaszczynski and Nower 2002), and many studies have shown that adolescent problem gamblers have erroneous beliefs about the independence of random gambling events and tend to overestimate their chances of winning (Delfabbro et al. 2006; Froberg 2006). Moreover, previous studies have found that problematic gambling behavior can be decreased with cognitive behavioral therapy (CBT) (Fortune and Goodie 2012). Therefore, some prevention programs attempt to address this lack of knowledge on the gambling odds and gambling-related misconceptions. For instance, Williams and Connolly (2006) examined the influence of increasing statistical and mathematical knowledge through classes focusing on gambling probabilities to reduce gambling participation among university students. A post-6-month evaluation showed that, after the intervention, students’ knowledge on gambling odds and on resistance towards gambling fallacies increased. However, this improved knowledge was not associated with observable changes in actual gambling behaviour. Donati et al. (2014) conducted a school-based prevention program among Italian high school students that targeted gambling-related knowledge and misconceptions, economic perception of gambling, and superstitious thinking. The results showed an improvement in correct knowledge about gambling and reduced misconceptions, perception of gambling’s profitability, and superstitious thinking. After the intervention, some behavioural changes were also reported (e.g., decrease in the percentage of gamblers and At-Risk/Problem gamblers).

More recently, Canale et al. (2016a) in a web-based gambling intervention among high-school students that focused on irrational gambling-related cognitions and misunderstandings related to randomness, found that the program was effective in decreasing gambling problems, but had no effect on gambling frequency and gambling expenditure.

Furthermore, Walther et al. (2013) conducted a prevention program for sixth and seventh grade students, that focused on gambling fallacies, signs of pathological gambling, and gambling features. The results showed that the program increased gambling knowledge and decreased problem gambling attitudes. However, the program had no significant influence on lifetime gambling.

Therefore, the effectiveness of school-based prevention programs based on unique determinants of behaviour have been found to show positive results in increasing gambling knowledge and modifying gambling misconceptions. However, the impact on actual gambling behaviour has yet to be established. This suggests that there is a lack of transference of knowledge towards behavioural change in gambling prevention initiatives.

On the other hand, the second group of prevention programs (i.e., comprehensive psychoeducational and skills training prevention programs), incorporate not only youth problem gambling risk and protective factors, but also more general risk and protective factors associated with adolescent risk behaviour, such as social-emotional and coping skills, problem-solving, decision-making and refusal skills, impulsivity, and sensation seeking. In fact, many studies have found that these variables can also play an influence in the development of problem gambling among youth (Barnes et al. 1999; Nower et al. 2004; Calado et al. 2017b).

In the literature, some programs have targeted variables associated with more general risk behaviours. For instance, Todirita and Lupu (2013) utilized an experimental design, in which children aged between 12 and 13 years were randomly assigned to three groups: a control group; an information group, in which children received information about gambling and gaming, and learned about gambling-related misconceptions; and a rational emotive education (REE) group, in which children learned how to enhance their emotional strength by making them aware of the irrational beliefs causing emotional distress and replacing them with rational and more adaptive beliefs. The results showed that the information group obtained more correct answers in questions related to misconceptions, illusion of control, attitudes, and cognitive errors. The authors concluded that using specific primary prevention tools for changing erroneous conceptions about games was more efficient than only using REE. This suggests the need to incorporate both gambling specific-related variables, and interpersonal and intrapersonal skills in prevention initiatives, and to focus on specific personality characteristics that can facilitate the development of these skills.

Williams et al. (2010) developed a school-based preventive program (the “Stacked Deck”) comprising five lessons that target gambling history, problem gambling, gambling fallacies, decision-making, and problem-solving skills. The findings of this study showed that students aged between 14 and 20 years improved their knowledge about gambling, gained better resistance to erroneous cognitions, but also improved problem-solving abilities and decision-making skills. Furthermore, after the intervention, participants reported significantly more negative attitudes toward gambling and showed a decline in problem gambling frequency. Similarly, Huic et al. (2017) developed another school-based gambling prevention program with Croatian adolescents, aged 14–17 years, which focused on cognitive distortions, knowledge of the nature of random events, as well as on other skills (such as problem-solving skills, refusal and self-efficacy). The results of the program showed a significant decrease in risk factors, more specifically a better knowledge about gambling and less gambling-related cognitive distortions. However, effects on problem-solving skills, refusal skills, and self-efficacy were not found. Moreover, the authors verified that the gambling frequency did not change in the experimental group as a result of the intervention. Therefore, these findings suggest that it is still unclear if preventive initiatives can improve skills and other characteristics more associated with general risky behaviours, and whether targeting these skills has the potential to protect young people from developing gambling-related problems.

The effectiveness of preventive initiatives that have focused on both types have not been well established and only a few studies have used this approach. However, broader and multidimensional approaches might be more important in order to ensure program effectiveness (Kourgiantakis et al. 2016). Some prevention programs have focused on generic skills, such as problem-solving skills, coping strategies, and self-efficacy (e.g., Williams et al. 2010) that have the potential to foster a healthy development among youth and prevent their engagement in problem gambling behaviour. However, to the present authors’ knowledge, no gambling prevention program has been developed that has incorporated modules concerning sensation seeking, a generic factor also associated with more general adolescent risky behaviour (e.g., Martins et al. 2008; Stautz and Cooper 2013). In fact, it has been found that sensation seeking prevents self-regulation and self-control, and higher sensation seekers tend to undervalue the dangers associated with risky behaviours (Hoyle et al. 2002). In addition, sensation seeking reaches a peak during adolescence, in which higher inclinations to seek excitement and relatively immature capacities for self-control, make adolescents more vulnerable to engage in risky behaviour (Steinberg et al. 2008). Furthermore, some research has suggested that adolescent risky behaviours when presented with hazardous behaviours that have both probable rewards and potential costs may be more sensitive in underestimating the costs and overestimating the rewards (Ernst et al. 2005). This highlights the need to target sensation seeking when designing youth interventions, in order to address novelty-seeking and to foster youth self-regulatory competence, through making the intervention more interactive, exciting and appealing—for increasing participant’s motivation and enhance their learning—but also through designing activities, in which adolescents are instructed about situations in which sensation seekers tend to respond in a significantly more risky manner and evaluate less the long term consequences of their behaviour (Donohew et al. 2000).

In fact, interventions focusing on sensation seeking have produced effects in the reduction of other risky behaviours in adolescents. For instance, Conrod et al. (2011) found that an intervention that targeted sensation seeking conducted among secondary students was effective in reducing problem drinking symptoms. Similarly, Zimmerman et al. (2007) examined the effect of a television campaign aimed at increasing safer sexual behaviour. The campaign utilized a sensation seeking targeting approach, in which creative messages that focused on high-sensation-seeking behaviour were used. The authors found that participants exposed to the campaign reported more increases in condom use, in comparison with the control group. Therefore, these promising results on other risky behaviours stresses the importance of utilizing a sensation seeking targeting approach (Palmgreen and Donohew 2010) for designing youth gambling prevention initiatives.

Moreover, the literature has emphasized the need to conduct more follow-up studies (National Research Council 1999; Kourgiantakis et al. 2016), in order to assess long-term (positive) effects and to monitor whether problem gambling develops during youths’ developmental life span. Additionally, there are a few studies that have tested the long-term stability of the preventive initiatives (e.g., Capitanucci et al. 2010; Ferland et al. 2005).

In addition, the style of delivery assumes particular relevance when designing preventive initiatives for young people. Research has emphasised that youth tend to respond well in programs that are interactive and engaging (Korn et al. 2006; Oh et al. 2017). Other studies highlighted the benefits of using multi-media learning to enhance the learning and retention of knowledge among youths (Ferland et al. 2002; Lavoie and Ladouceur 2004). Moreover, interactive sessions that encourage engagement and discussions are more successful in changing performance among youth (Davis et al. 1999). Korn et al. (2006) who developed a program comprising interactive games, in which students learned about time and money management, general risk perception, decision-making, and the concept of randomness, showed that participants enjoyed the program’s interactivity, and reported gaining knowledge and awareness about gambling. Furthermore, prevention programs delivered by specialized staff have been found to be more effective than the ones conducted by untrained teachers (e.g., Donati et al. 2014; Todirita and Lupu 2013), which highlights the importance of training prevention program providers and developing preventive initiatives with a solid theoretical foundation.

To summarize, school-based programs constitute an important component of an overall problem gambling prevention strategy. However, there is a lack of published research on the design, development and evaluation of original prevention programs, based on a solid theoretical evidence (St-Pierre and Derevensky 2016). To date, very few preventive initiatives have been evaluated. In addition, some of the prevention programs that have been evaluated have obtained reliable improvements in gambling knowledge, knowledge about problem gambling, and a decrease in gambling fallacies, but most of these programs have not shown any actual behavioural change. However, improvements in gambling knowledge, misconceptions, and attitudes constitute relevant outcomes in a prevention program, but are of limited importance if they are not accompanied by behavioural change. Moreover, there are few prevention programs that have incorporated both problem gambling risk and protective factors, as well as general risk and protective factors for adolescent risk behaviour. Although some prevention programs have focused on generic variables, to the present authors’ knowledge there are no youth gambling prevention programs that have targeted sensation seeking, and that have evaluated this variable. Furthermore, there are few prevention programs that have conducted a follow-up.

Consequently, based on existing empirical evidence, the main goal of the present study was to evaluate a new youth gambling prevention program that was developed in Portugal following the theoretical principles of prevention and based on CBT models for reducing problematic gambling behaviour, and by adopting a sensation seeking targeting approach when designing preventive initiatives for youth risky behaviours. The specificity of this intervention consisted of (1) increasing correct knowledge and reducing gambling-related misconceptions, (2) decreasing gambling behaviour, (3) producing a change in factors associated with adolescent risky behaviours in general, such as reducing the levels of sensation seeking, and promoting a more optimal level of experiencing intense, novel and arousing stimuli. The intervention attempted to help students to improve their decision-making skills, when faced with situations that might encourage sensation seekers to respond in a riskier manner and consider only their immediate gratification, which might promote their engagement in more unsafe and dangerous behaviours. The aim was to evaluate the program’s short-term effects, but also the long-term effects by conducting a follow-up after the intervention. Therefore, in line with the aforementioned goal, and with the specific characteristics of this intervention, it will be hypothesised that (1) compared to the control group, the students who will receive the intervention will report at post-test, more correct knowledge about gambling, and a reduction in gambling-related misconceptions, lower rates of problematic gambling, gambling frequency, amount of money spent, total hours spent on gambling per week, more realistic attitudes towards gambling, and lower levels of sensation seeking, and (2) these changes will remain stable after a 6-week follow-up.

## Methods

### Participants

The present sample comprised 111 high-school students (65 females, 46 males; mean age = 17.64 years; SD = 1.62) enrolled in a professional school in Lisbon (Portugal). Written informed consent was requested from students (and their parents in the case that students were minors), and they were assured that the data provided would be handled confidentially. This study was conducted during normal school time.

### Measures

#### Demographics and Gambling Behaviour

Demographic questions collected information about age and gender. Participants were also asked to indicate how often they had gambled during the past 2 months (from 1 “Never” to 7 “Everyday”), how many hours they gambled during the week, and the most amount of money they have ever spent on gambling from 1 (“Never bet money on gambling”) and 5 (“Between €100 and €1000”).

#### Questionnaire of Misconceptions and Knowledge About Gambling (Ferland et al. 2002)

This instrument consists of two sections—knowledge about gambling, and gambling-related misconceptions. The scales comprise 16 items using a 4 Likert point scale ranging from 1 (totally disagree) to 4 (totally agree). Nine items assess knowledge of gambling (yielding a maximum score of 36) and seven items assess misconceptions of gambling (yielding a maximum score of 28). The following are examples of items targeting knowledge and misconceptions respectively: “Buying lottery tickets is a type of gambling” and “When I’m betting, I must know the tricks and strategies if I want to win”. This scale has already been used in many studies, and previous analysis has always demonstrated a two-factor structure. The first factor corresponds to knowledge about gambling, and the second factor corresponds to misconceptions about gambling (Ferland et al. 2002; Ladouceur et al. 2004). For the present study, the scale was translated and back translated to the Portuguese language, and exploratory factorial analysis also demonstrated a two-factor structure in this sample. The reliability was considered adequate (knowledge about gambling: α = 0.70 at pre-test; α = 0.76 at post-test; α = 0.71 at follow-up; gambling related misconceptions: α = 0.62 at pre-test; α = 0.76 at post-test, α = 0.75 at follow-up).

#### DSM-IV-Multiple Response-Juvenile (DSM-IV-J-MR, Fisher 2000)

This instrument assesses youth problem gambling. It comprises nine questions, which were developed for adolescents and modelled on the DSM-IV criteria for pathological gambling (e.g., “How often have you found yourself thinking about gambling or planning to gamble”; “After losing money gambling, have you returned another day to try and win back money you lost?”). Participants who obtain a score of 0 or 1 are classified as social gamblers, a score of 2 or 3 indicates at-risk gambling, and a score of 4 or higher is indicative of problem gambling. The DSM-IVMR-J has good construct validity in terms of its ability to reliably distinguish between social and pathological gamblers (Fisher 2000). This scale was previously validated in Portuguese samples (Calado et al. 2016), which confirmed the one-dimensional structure of the original scale, and showed good criterion validity. For the present study, the reliability of this scale was considered satisfactory (α = 0.77 at pre-test; α = 0.66 at post-test; α = 0.71 at follow-up). Due to the relative brevity of the intervention and the relative shortness of time between the pre-test, post-test, and follow-up, when completing the DSM-IV-J-MR, participants were asked to refer to the past 2 months (and not to the last 12 months, which is the normal timeframe of this scale) to investigate changes in problem gambling behaviour.

#### Attitudes Towards Gambling Scale (ATGS8, Wardle et al. 2011)

The ATGS8 is an instrument that was developed for the 2010 British Gambling Prevalence Survey to assess attitudes towards gambling for people aged 16 years and older (e.g., “People should have the right to gamble whenever they want”; “Gambling should be discouraged”). The scale comprises eight items with responses given on a 5-point Likert scale. Higher scores indicate more positive attitudes towards gambling. Previous validations of this scale confirmed its one-dimensional structure, and good concurrent validity (Canale et al. 2016b). For the present study, the instrument was translated and back translated to the Portuguese language. In the present study, the reliability of this scale was considered satisfactory (α = 0.76 at pre-test, α = 0.76 at post-test, α = 0.66 at follow-up).

#### Brief Sensation Seeking Scale (BSSS, Hoyle et al. 2002)

This instrument assesses sensation seeking, and comprises eight items (e.g., “I would like to explore strange places”; “I like wild parties”). The items are responded to on a 5-point Likert-type scale ranging from 1 (strongly disagree) to 5 (strongly agree). Higher scores indicate higher levels of sensation seeking. This instrument has been extensively used with adolescents and emerging adults. Moreover, this scale has already been previously validated with Portuguese samples (Chitas 2010) demonstrating adequate psychometric properties. For the present study, the Portuguese version validated by Chitas (2010) was administered, and its reliability was considered adequate (α = 0.84 at pre-test, α = 0.77 at post-test, α = 0.76 at follow-up).

### Procedure and Design

In order to evaluate the efficacy of the intervention and to evaluate changes in the variables considered in the present study over time as a function of the treatment condition, an experimental design was conducted with two groups (experimental vs. control) and two measurements (pre-test and post-test sessions). For the present study, six classes participated, which were randomly assigned to the experimental and control groups. Random assignment of classrooms to each condition was accomplished by using a random number table.

The experimental group completed the measures described above 1 week before the intervention (pre-test), at the end of the last session of the program (post-test), and 6 weeks after the intervention has ended (follow-up). The control group was administered the pre-test and post-test questionnaires (within the same week of the experimental group), but did not receive the intervention. While the experimental group received the intervention, the control group continued with the normal school activity. Pre- and post-intervention as well as follow-up assessments were collected in the classroom via pen and paper.

Informed written consent for participation was obtained from both parents and children. All participants were informed about the nature of the study, and research objectives of the data, with assurances that non-participation would not lead to negative consequences. It was also explained that responses to the questionnaires were anonymous and confidential. Only the respondents’ birthday, mother’s first name, and the last two digits of their telephone number were required to match pre-test questionnaires to post-test and follow-up surveys. The structure of the program was given ethical approval by the research team’s university ethics committee.

### The Intervention

The intervention comprised a primary prevention program, and was designed by the first author with constructive input from the remaining authors. The intervention was delivered by the third and fourth authors, who had received intensive training concerning youth gambling and school prevention. The trainers had received regular supervision from the first author. Each trainer stayed in the same class throughout the whole intervention to avoid contamination across conditions and to develop continuity in the relationships between trainers and students. The nature and content of the intervention was derived from the previous literature about youth gambling, more specifically variables that are known to contribute to the initiation of gambling behaviour, and for the development of gambling-related problems among this age group. The specificity of the intervention comprised didactic units to increase correct knowledge and reduce gambling-related misconceptions, but also to target on other factors associated with adolescent risky behaviours in general (e.g., reducing the levels of sensation seeking, and helping adolescents to practice other abilities during the intervention, such as helping students to improve their decision-making skills when faced with situations that might encourage more riskier responses). A variety of methods and techniques to deliver the activities to the students were used, including: (a) icebreaking and warm-up activities to make the students feel comfortable with the trainer of the program and enhance group cohesiveness; (b) quizzes in order to facilitate previously learned content; (c) interactive methods such as live discussions, and real-life situations in which students could practice newly learned skills; (d) encouragement of critical thinking, especially to promote more insight when deciding to engage in a risky behaviour, and to reduce the levels of sensation seeking; (e) team learning—in pairs, threes, small groups; (f) examples from life-real situations; (g) positive atmosphere building so that students could feel comfortable in discussing topics related to training activities. These techniques were also used to increase students’ engagement in the program.

The program comprised five didactic units, each consisting of one session, that were delivered in class during normal school time. The intervention was delivered on a weekly basis and each session lasted approximately 1 h. The program started in the first week of December 2016, then had a small break (for Christmas) and restarted again in the first week of January 2017. The first session of the program started with an icebreaking activity in order to create a positive atmosphere in the class and for the students to feel safe. The other sessions each started with an interactive quiz which summarized and recapped the content delivered in the previous sessions. Detailed explanations of each didactic unit are outlined in Table 1.

**Table 1:** Detailed description of the prevention program

Session	Didactic unit	Description
Session 1	(“Gaming or Gambling”?)	This session began with an icebreaking activity to introduce the program, and make the students feel comfortable with the facilitator. The students were then asked to form teams, and to work in groups. Then, an activity was carried out in order to help students distinguish between gambling and gaming. Each team was presented with images of videogames and gambling activities, and were asked to indicate which of them were gambling. Each team had to compete with each other in order to give the most correct answers. After the presentation of these images, the concept of gambling was introduced, and students were made aware that gambling involves betting money or something of material value on an event with an uncertain outcome, and that humans are unable to control
Session 2	Cognitive distortions and gambling-related misconceptions	This session began with a quiz to recap the main concepts that were covered in the previous session. Then, one student, who was randomly selected in the class, was asked to play roulette, and to show the group the winning number. The other students in the class were asked to form teams, each one comprising four or five members. Secondly, the same student was asked to play roulette again, and to show the group the winning number again. After the second play, each team (based on the outcome of the first two bets) was asked to make their own bets and write their bets on a sheet. Then, the facilitator gave the sheet to the roulette player, who was asked to read aloud the bet of each team, and to again play roulette to see if the number which each team had bet would come up in any of the plays. This activity was followed by a discussion, in which the concept of independent random events was introduced, and it was explained that it was not possible to predict any gambling outcome based on previous plays
Session 3	Attitudes towards gambling and money	This session began with a quiz. The quiz in this session was longer, as this was the first session after the Christmas break, and it aimed to recap the content of the two previous sessions. After the quiz, the students were shown a picture of a young male gambler who went to Las Vegas. After looking at the picture, students were given a short description about the young man. They were told that he was a Portuguese man who used to gamble in his spare time, and that he said that gambling is a very exciting activity. The students were then asked to work in groups again, and to write down on a sheet their opinions, thoughts, and feelings about people who gamble, and if they thought that individuals who gambled had specific characteristics that individuals who did not gamble did not have. After each team wrote their thoughts and opinions, the facilitator collected the sheets, and read loud what each team had written. This activity was followed by a brief discussion, in which the thoughts that students had about gambling were debated. It was noted that there were some stereotypes and labels regarding gamblers, such as being categorized as cool, or having certain traits, but there were gamblers across all ages, and from many social and cultural backgrounds, and it could be a bias to have a stereotype towards people who gamble. After this activity, the students were shown another picture of the same young man, and they were told that he had won 500 euros in one evening in the casino. Then, the students were asked the following questions “Do you think that it is nice to win 500 euros in one evening? Do you think that people who win too much money in a very short period are happier? Do you think that there are other things in life that can make people happy?” After these questions, the students were given a second sheet, in which they were instructed to write about how the day of a happy person. This activity was followed by a final discussion, in which it was concluded that there are important things in life besides money, and that sometimes people who gamble want to win money in a very easy way, but most of the time this will not happen (because it is not possible to predict the gambling outcomes). At the end of the session, students were given a questionnaire about attitudes towards gambling to reflect on their own personal attitudes, and they were told that they were free to share their responses with the rest of the class, if they felt comfortable in doing so
Session 4	Sensation seeking	This session began with a quiz to recap the concepts that were covered in the previous session. After the quiz, the students formed teams, comprising three or four members, and each team had to rank activities that were presented on a PowerPoint slide in terms of the level of sensation seeking that each activity provokes, on a scale from 1 (the activity does not provoke any sensation seeking) to 5 (the activity provokes a high level of sensation seeking and excitement). The activities shown were bungee jumping, taking a trip with no pre-planned routes or timetables, climbing Mount Everest, driving under the influence of alcohol, sleeping in, walking in a dark street where there are no people, going out with people that they have just met, talking on a mobile phone, skipping school, smoking cigarettes, buying a lottery ticket, gambling on bingo, and gambling at an online casino. While each team was ranking each activity, the facilitator wrote the ranks of each team on a sheet, and in the end of this activity, the students were told which activities had the highest score. Then, the students were asked to fill in a sheet for the activities that had the highest score for excitement, adrenaline, and sensation seeking, as well as the three gambling activities. On this sheet, the students had to write down the stimuli, thoughts, feelings, short-term consequences, long-term consequences, and final decision for engaging in this activity. This activity was followed by a dynamic discussion, in which it was explained that there are some activities that cause some high levels of adrenaline, and excitement, but that these feelings were short-lived. In addition, it was discussed that in life when we take a decision if we engage in a specific activity, we need to think about both long-term and short-term consequences. Finally, the facilitator gave the students another sheet, which outlined the steps that people need to take in order to make mature and mindful decisions about engaging in an activity or behaviour
Session 5	Problem gambling	This session began with a quiz in order to briefly recap the concepts that were covered in the previous session. The students were then shown a video of the actor Ben Affleck (known for his gambling addiction) in front of a poker table, and facing another gambling loss. After the video, the students were presented with a newspaper headline which informed them that the gambling behaviours of this actor had affected his marriage with the actress Jennifer Garner. This article added that the couple travelled to Las Vegas, and that Ben bet thousands of dollars, and although Jennifer was very upset with his behaviours, he claims that he could not stop his behaviour. According to this source, Ben mentioned that he felt bad or fed up when trying to cut down his gambling behaviour, and that the actor had been accused of fraud because he had tried card-counting to win playing blackjack. After the video and the newspaper headline, the students were told that the actor was known for his gambling addiction, and then they were asked what aspects mentioned in the video and in the newspaper article that indicated the actor had developed a gambling addiction. This activity was followed by a discussion, in which students were shown in a PowerPoint slide the criteria of problem gambling according to DSM. During this activity, it was explained that for someone being considered a problem gambler, they must show at least four of the criteria. After this, the students were asked to work again in groups, and each group was given a document that showed cases of adolescents with problem gambling behaviour. These cases were taken and adapted from Griffiths (1995). The students were asked to identify the signs and criteria of problem gambling that were present in each of the cases. At the end of this session, the students were given tips about responsible gambling, and were provided contacts concerning relevant institutions that provided support for problem gambling in Portugal

#### Data Analysis and Analytic Strategy

In the first step, all variables were examined for skewness and kurtosis at pre-test, post-test, and follow-up assessments. The variable “amount of hours gambled in the week” was found to be skewed in pre-test, post-test, and follow-up, and a logarithmic transformation was used to normalize the distributions (Tabachnick and Fidell 2007). In a second step, the baseline equivalence between the experimental and control groups was tested with independent sample t-tests for continuous variables and with Chi square tests for categorical variables. Then, the short-term efficacy of the intervention was analysed by conducting a mixed 2 × 2 ANOVA with *time* (pre-test and post-test) as within-factor and *group* (experimental and control) as between-factor for each dependent variable. As a fourth step, the short-term effects on the basis of problem gambling severity were assessed. Inside the experimental group, a mixed 2 × 2 ANOVA was conducted with *time* (pre-test and post-test) as within-factor and *problem gambling severity* (non-problem and at-risk/problem) as between-factor for each dependent variable for which significant interaction effects were found in the previous analysis step. Subsequently, the long-term efficacy was analysed by considering results from the experimental group. More specifically, paired sample t-tests were used to compare post-test and follow-up scores for the dependent variables for which the intervention was found to be effective at post-test. Finally, using McNemar tests, we compared the percentage distribution of At-Risk/Problem gamblers respectively found from post-test to follow-up in the experimental group. In line with previous gambling prevention initiatives (e.g., Donati et al. 2014), in the present study, problem gambling severity was categorized as the combination of at-risk and problem gambling, and therefore individuals who obtained a score of 2 or higher in the DSM-IV-J-MR instrument were considered to belong to the problem gambling group and were compared against non-problem gamblers (those who obtained a score of 0 or 1).

## Results

### Baseline Equivalence Evaluation

As a first step, the equivalence was tested between the experimental and the control group for socio-demographic data, for the variables considered in the intervention, and for problem gambling behaviour. These analyses were conducted with the students of the experimental group who took part in the pre-test, attended the full intervention, and completed the post-test (n = 56), and with the participants from the control group who completed the pre-test and post-test measures (n = 55). No significant differences were found between the two groups with regard to socio-demographic variables, gambling frequency, amount of money spent gambling, total hours spent gambling per week, attitudes towards gambling, correct knowledge and misconceptions about gambling, sensation seeking, and problem gambling behaviour as assessed at the pre-test session (see Table 2).

**Table 2:** Baseline equivalence evaluation between the experimental group (n = 56) and the control group (n = 55)

	Experimental group		Control group		T	*p* value
	Mean	SD	Mean	SD		
Age	17.55	1.60	17.73	1.66	− 0.56	n.s.^a^
Gambling frequency	2.57	1.82	2.11	1.52	1.45	n.s.
Amount of money spent	2.34	1.25	1.95	1.16	1.72	n.s.
Total hours	0.16	0.27	0.12	0.22	1.03	n.s.
Attitudes	24.02	3.64	23.76	4.47	0.33	n.s.
Knowledge	21.38	2.95	21.89	3.41	− 0.85	n.s.
Misconceptions	17.16	2.31	16.25	3.03	1.77	n.s.
Sensation seeking	3.47	0.79	3.45	0.85	0.12	n.s.

	N (M)	N (F)	N (M)	N (F)	Chi square	*p* value
Gender	24	32	22	33	0.09	n.s.

	N (NPGs)	N (ARPGs)	N (NPGs)	N (ARPGs)^b^		
Problem gambling behaviour	44	12	50	5	3.25	n.s.

^a^Non-significant (n.s.)

^b^Non-problem gamblers (NPGs) versus at-risk/problem gamblers (ARPGs). Non-problem gamblers (NPGs) were categorized as having a score of 0 or 1 and at-risk/problem gamblers (ARPGs) as having a score of 2 or higher in the DSM-IV-J-MR instrument

### Short-Term Efficacy Evaluation

Short-term intervention effects were tested performing a mixed 2 × 2 ANOVA with *time* (pre- and post-test) as within-factor and *group* (experimental vs. control group) as between-factor. The analyses were performed on each of the following dependent variables: gambling frequency, amount of money spent, total hours spent gambling per week, correct knowledge about gambling, misconceptions about gambling, attitudes towards gambling, and sensation seeking. The main effect of interest was the *group* x *time* interaction.

Significant interactions were found for total hours spent gambling per week [F(1,109) = 4.8, *p* < 0.05, ηp^2^ = 0.04], knowledge [F(1,109) = 48.91, *p* < 0.001, ηp^2^ = 0.31], gambling-related misconceptions [F(1,109) = 59.55, *p* < 0.001, ηp^2^ = 0.35], and attitudes [F(1,109) = 14.91, *p* < 0.001, ηp^2^ = 0.12]. Interactions were non-significant for gambling frequency [F(1,109) = 3.37, *p* = 0.07], amount of money spent gambling [F(1,109) = 0.69, *p* = 0.41], and sensation seeking [F(1, 109) = 2.89, *p* = 0.09].

Post-hoc t-tests were conducted for those variables for which significant interactions were found. The post hoc t-tests showed the interaction effects to be due to significant changes from pre-test to post-test in the experimental group but not in the control group. More specifically, in the experimental group there was a significant improvement in correct knowledge about gambling and a significant reduction of gambling-related misconceptions, attitudes towards gambling, and total hours spent on gambling per week (see Table 3).

**Table 3:** Mean scores compared with paired-samples t test (and related effect sizes) for the experimental group (n = 56) and the control group (n = 55) at pre- and post-test for the variables with significant interaction

	Pre-test		Post-test		*T*	d
	M	SD	M	SD		
*Knowledge about gambling*						
Experimental group	21.38	2.95	26.21	3.52	− 8.82***	1.18
Control group	22.07	3.41	22.56	3.67	− 1.72 (n.s.)	–
*Misconceptions*						
Experimental group	17.16	2.31	12.45	2.86	9.56***	1.28
Control group	16.25	3.03	15.93	3.01	1.18 (n.s.)	–
*Attitudes*						
Experimental group	24.02	3.65	21.21	4.08	4.11***	0.55
Control group	23.76	4.47	23.93	4.17	− 0.48 (n.s.)	–
*Total hours*						
Experimental group	0.16	0.27	0.08	0.17	2.43*	0.32
Control group	0.12	0.22	0.12	0.19	(n.s.)	–

*n.s.* non-significant

*p < 0.05; **p < 0.01; ***p < 0.001

### Short-Term Efficacy Evaluation Based on Problem Gambling Severity

In order to verify if the aforementioned short-term effects were obtained by all students in the experimental group regardless of their problem gambling severity status, students without gambling problems (classed as non-problem gamblers), and students with gambling problems (classed as at-risk/problem gamblers) were separately analysed inside the experimental group. Therefore, a mixed ANOVA with *time* (pre-test and post-test) as within-factor and *problem gambling severity* (non-problem and at-risk/problem) as between-factor was performed on each dependent variable for which the interactions were found to be significant (more specifically, knowledge about gambling, misconceptions, attitudes, and total hours). No significant interactions were found for attitudes [F(1,54) = 0.23, *p* = 0.63]; correct knowledge about gambling [F(1,54) = 0.39, *p* = 0.54], and misconceptions [F(1,54) = 0.02, *p* = 0.90]. However, a significant interaction was found for total hours spent gambling per week [F(1,54) = 24.71, *p* < 0.001, ηp^2^ = 0.31]. This suggests that for this specific variable, a different pattern of change between pre-test and post-test occurred in the two types of gamblers. However, the main effect of time was significant for knowledge [F(1,54) = 38.49, *p* < 0.001, ηp^2^ = 0.42], misconceptions [F(1,54) = 61.53, *p* < 0.001, ηp^2^ = 0.53], attitudes [F(1,54) = 9.43, *p* < 0.05, ηp^2^ = 0.15], and total hours spent gambling per week [F(1,54) = 27.31, *p* < 0.001, ηp^2^ = 0.34], which suggests that a significant change had occurred from pre-test to post-test in all these variables.

Post-hoc t-tests showed that significantly higher levels of correct knowledge about gambling occurred from pre-test to post-test in both types of gamblers. In addition, gambling-related misconceptions significantly improved from pre-test and to post-test in both types of gamblers. However, a significant decrease in attitudes towards gambling from pre- to post-test only occurred among non-problem gamblers, suggesting that the intervention was unsatisfactory in reducing attitudes towards gambling among the problem gambling group (see Table 4).

**Table 4:** Mean scores compared with paired-samples t-test (and related effect sizes) for non-problem gamblers (n = 44) and at-risk/problem gamblers (n = 12) in the experimental group

	Pre-test		Post-test		*T*	d
	M	SD	M	SD		
*Total hours*						
Non-problem gamblers	0.06	0.14	0.05	0.13	0.34 (n.s.)	–
At-risk/problem gamblers	0.54	0.28	0.19	0.23	3.74*	1.08
*Knowledge about gambling*						
Non-problem gamblers	22.34	3.01	26.23	3.03	− 6.64***	1
*Misconceptions*						
At-risk/problem gamblers	21.42	3.12	26.17	5.10	− 2.97*	0.86
Non-problem gamblers	17	2.44	12.32	2.67	8.74***	1.32
At-risk/problem gamblers	17.75	1.71	12.92	3.55	3.87**	1.12
*Attitudes*						
Non-problem gamblers	23.77	3.78	20.80	4.28	3.69**	0.56
At-risk/problem gamblers	24.92	3.06	22.75	2.86	1.79 (n.s.)	–

*n.s.* non-significant

*p < 0.05; **p < 0.01; ***p < 0.001

### Long-Term Efficacy Evaluation

In order to evaluate the long-term efficacy of the intervention, paired sample t-tests were conducted for the post-test and follow-up-test scores of the variables for which the intervention was found to be effective in the short-term (knowledge about gambling, misconceptions, attitudes, and total hours spent gambling per week). A total of 39 participants completed the post-test and the follow-up sessions. The results of the t test showed no significant differences between the post-test and follow-up scores, suggesting the permanence of the intervention effects over some time for correct knowledge about gambling, misconceptions about gambling, attitudes towards gambling, and total hours spent gambling per week (see Table 5).

**Table 5:** Mean scores compared with paired-samples t-test for the training group (n = 39) at post-test and follow-up

	Post-test		Follow-up		T
	M	SD	M	SD	
Knowledge about gambling	26.49	3.10	25.59	3.22	1.64 (n.s.)
Misconceptions	12.46	2.71	12.74	2.93	− 0.68 (n.s.)
Attitudes	21.08	3.59	20.87	3.69	0.64 (n.s.)
Total hours	0.07	0.17	0.09	0.17	− 0.92 (n.s.)

*n.s.* non-significant

### Problem Gambling Behaviour

To verify that the intervention had the desired effect on adolescent self-reported problematic gambling, changes in the percentage of at-risk/problem gamblers inside the experimental group from pre-test to follow-up were evaluated using a McNemar’s test. The percentage of at-risk/problem gamblers decreased from 21.4% in pre-test to 7.7 in follow-up. The exact McNemar’s test determined that this difference was statistically significant (*p* < 0.05).

## Discussion

To the authors’ knowledge, the present study is the first gambling prevention program conducted in Portugal. At the same time, the intervention attempted to overcome some of the limitations of previous international programs, such as the lack of follow-up, and the lack of assessment of behavioural change. In addition, the program attempted to incorporate both unique and common determinant approaches into the prevention program due to the benefits of both approaches, and to fill a gap in the gambling prevention literature, because very few studies have included both components (i.e., gambling knowledge and general factors in the program design, and evaluation).

Overall, the findings demonstrated that the intervention produced the desired effect on knowledge about gambling, gambling-related misconceptions, attitudes towards gambling, and total hours spent gambling per week. More specifically, students who had received the intervention enhanced their knowledge about gambling, and reduced their gambling-related misconceptions, attitudes towards gambling, and the total hours spent gambling per week, whereas the students from the control group did not show a significant change on these variables from pre-test to post-test. The results obtained in the experimental group were in line with previous preventive initiatives found in the literature (e.g., Donati et al. 2014; Taylor and Hillyard 2009; Ferland et al. 2002). Nevertheless, the intervention did not show any effect on gambling frequency, amount of money spent gambling, which confirm other prevention programs (Williams et al. 2010; Huic et al. 2017).

The results obtained for the amount of money spent and gambling frequency may be explained by the fact that very few students reported that they spent the highest amounts of money on gambling in the survey (between €100 and €1000); and only relatively few students reported gambling at the highest frequencies. In addition, the results obtained for sensation seeking may be related to the fact that effects on personality characteristics and intrapersonal skills were unlikely to occur during the time of the evaluation of this program (post-test and follow-up). In fact, research suggests that sensation seeking is unlikely to be reduced via interventions (Zuckerman 2007), but this does not imply that sensation seeking should not be incorporated into interventions aimed at reducing risky behaviour during adolescence. In fact, it is possible to change how sensation seeking is expressed, minimizing unhealthy expressions (Arnett 1995). Studies have demonstrated that personality-targeted interventions, especially sensation seeking, produce long-term effects in the reduction of other risky behaviours in adolescents, including alcohol use (Conrod et al. 2011), marijuana use (Palmgreen et al. 2001), and promoting safer sexual practices (Zimmerman et al. 2007). In fact, a growing body of research has documented that interventions designed to address the needs for novelty and sensation seeking considerably enhance the ability to capture the attention of individuals likely to engage in health-risky behaviours, as well as their motivation to change their behaviour (Donohew et al. 2000; Lorch et al. 1994).

The short-term changes on correct knowledge concerning gambling, and gambling-related misconceptions were found in both problem- and non-problem gambling adolescents. However, the intervention only reduced the total hours spent on gambling per week among the problem gambling group. This finding may be explained by the fact that the problem gambling group reported gambling more hours per week in comparison with the non-problem gambling group. Moreover, the intervention only reduced the attitudes towards gambling among the non-problem gambling group. This result may be explained by the self-perception theory (Bem 1967), which postulates that individuals typically conjecture about the attitudes they have based on their own behaviour, without any previous disagreeable feelings. Therefore, problem gamblers would likely show more resistance to change their attitudes because they are in consonance with their behaviour. On the other hand, the Integrated Behavior Model (Montaño and Kasprzyk 2008) postulates that attitudes and subjective norms regarding a behaviour are based on social acceptance of family members, friends or significant others. Moreover, some research has found that the development of more positive attitudes towards gambling is associated with gambling approval by close others, which in turn is associated with more gambling involvement (e.g., Hanss et al. 2014; Orford et al. 2009). Therefore, future interventions should explore the relationships that adolescents have with significant others who gamble (e.g., friends and family), the outcomes of their gambling behaviour, beliefs about whether significant others think that adolescents will engage in this behaviour, and focus on peer resistance/refusal skills.

Regarding long-term-efficacy, the results showed that post-test mean scores of the variables for which the intervention was found to be effective in the short-term, did not differ significantly from the follow-up ones, indicating stability of the effects over a period of 6 weeks for participants who have attended the training program. This represents an important finding, because it indicates that the content of this program was suitable for the target population and delivered in a manner that allowed for some retention of the material.

Furthermore, in relation to the effects on problem gambling behaviour, the findings showed that among students from the experimental group, there was a significant reduction in the percentage of at-risk/problem gamblers from pre-test to follow-up. This finding appears to satisfy one of the main goals of a preventive program, that is, to prevent vulnerable individuals, such as adolescent gamblers, from the development of severe gambling problems (Dickson-Gillespie et al. 2008). In fact, this result is of particular relevance, because among previous prevention programs that have conducted a behavioural efficacy evaluation, only Williams et al. (2010), Donati et al. (2014) and Canale et al. (2016a) have reported positive results. In the present study, the effect on behavioural efficacy only occurred in relation to problem gambling behaviour and not in gambling frequency (although this was very close to achieving statistical significance), which confirms findings from previous gambling prevention programs (e.g., Canale et al. 2016a). One possible explanation for this is that students with the most symptoms of problem gambling may profit more from prevention initiatives (Williams et al. 2010).

Despite these encouraging findings, the present intervention study is not without its limitations. Firstly, the follow-up was conducted only 6 weeks after the end of the intervention, which prevented a longer-term evaluation of the results. However, due to school constraints, and the impending exams period, it was not possible to conduct a longer follow-up after the end of the intervention. Therefore, future intervention studies should attempt to conduct longer follow-up periods in order to draw more solid conclusions concerning the effects obtained in the intervention. In addition, both the experimental and the control group had modest sample sizes, and not all students were present during the follow-up, which made the sample size even smaller. However, power analysis revealed that this sample size was adequate to conduct the statistical analysis used in this paper, and to obtain effect sizes. Furthermore, the sample size obtained was similar to other previously published prevention programs (e.g., Todirita and Lupu 2013). Furthermore, the information obtained concerning the behaviour of the adolescents was based on self-reports, which might have led to some measurement errors and/or biases. Therefore, further research with larger sample sizes and more objective measures of gambling and gambling-related variables, such as the Iowa Gambling Task, and the Gambler Fallacy Task (Primi and Chiesi 2011) could be utilised.

The findings of this study have potential important implications. Firstly, the present study provides support for the integration of interventions aimed at promoting more responsible gambling within high-school curricula. Adolescence is characterized by an increase in risky behaviours, and by a limited developed cognitive ability, which makes this age group more vulnerable to gambling hazards (Oh et al. 2017). In fact, this program was effective in improving correct knowledge about gambling and in improving gambling-related misconceptions, and early evidence suggests that correcting irrational gambling-related cognitions can lead to behavioural change (Delfabbro 2004). Consequently, brief school-based prevention programs, easy to disseminate for large group of students that teach students about independence of random events in gambling games, and illusion of control features, may be recommended.

In conclusion, the results of this study provide an important contribution to the emergent body of literature concerning youth gambling prevention programs, by presenting an original program designed and evaluated in Portugal. Overall the results attested the short and long-term effectiveness of an integrated intervention in increasing correct knowledge of gambling, and in reducing gambling related misconceptions both among non-problem and at-risk/problem gamblers, and also demonstrated some changes in problem gambling behaviour. School-based gambling prevention initiatives are still limited in the literature, and the present study reinforces the need for developing more interventions and provides important suggestions for future programs to improve its content and subsequent effects.
